# A Room for Long-Lived Plasma Cell Contribution in Immune Cytopenias?

**DOI:** 10.3390/cancers17091537

**Published:** 2025-05-01

**Authors:** Tricia Don, Manisha Gadgeel, Süreyya Savaşan

**Affiliations:** 1 Pediatric Residency, Children’s Hospital of Michigan, Detroit, MI 48201, USA; tdon@dmc.org; 2 Hematology/Oncology Flow Cytometry Laboratory, Children’s Hospital of Michigan, Detroit, MI 48201, USA; mgadgee@med.wayne.edu; 3 Department of Pediatrics, Central Michigan University College of Medicine, Saginaw, MI 48602, USA; 4 Division of Hematology/Oncology, Pediatric Transplantation and Cell Therapy Program, Children’s Hospital of Michigan, Detroit, MI 48201, USA; 5 Barbara Ann Karmanos Cancer Center, Detroit, MI 48201, USA; 6 Department of Pediatrics, Wayne State University School of Medicine, Detroit, MI 48201, USA

**Keywords:** immune cytopenia, Evans syndrome, long-lived plasma cells, B cell development, autoimmune disease, autoantibodies, epigenetics, cancer

## Abstract

Immune cytopenias, such as autoimmune hemolytic anemia, immune thrombocytopenia, and Evans syndrome, are characterized by autoantibodies targeting various blood cells, initiating their destruction. Interactions between T cells, B cells, their ultimate maturational plasma cell descendants, dendritic cells, and macrophages result in antibody production, including the autoreactive ones. Autoimmune phenomena can be idiopathic or associated with various immune dysregulation conditions or malignancies. Interventions disrupting this complex network at different levels have been used to treat immune cytopenias with certain levels of success. Some cases are known to be refractory to many different therapeutic approaches, including the ones eliminating B cells. In some such cases, targeting plasma cells resulted in disease control. Among plasma cell compartments, unique long-lived plasma cells (LLPCs) residing primarily in the bone marrow, are specialized antibody-producing cells with an extended lifespan, capable of persistently secreting antibodies. LLPCs can evade conventional therapeutic strategies designed to target often-proliferating cells. Research focusing on the role of LLPCs in autoimmune phenomena including immune cytopenias has provided evidence for their role, characterized by the sustained production of autoantibodies. Frequent genetic mutations and progression to other immune dysregulation entities have been reported in a group of children with immune cytopenias. This might provide new insights focusing on the potential underlying genetic and epigenetic mechanisms leading to generation and maintenance of LLPCs in autoimmune disorders. We provide a brief review of LLPC biology and evidence for their role in immune cytopenias with potential future implications in this article.

## Simple Summary

This review will start by discussing an interesting case seen in our clinical practice. Following this, it will focus on a brief overview of humoral immunity physiology, including B and plasma cell development, with eventual emphasis on long-lived plasma cells. A concise focus on the biology of long-lived plasma cells with their potential contribution to autoimmunity in general, and more specifically to autoimmune cytopenias including Evans syndrome, will then ensue. The review will conclude with the association of autoimmune phenomena and cancer, as well as potential genetic and epigenetic links to long-lived plasma cell function. The aim of the article is to introduce the potential role of long-lived plasma cells in autoimmune cytopenia to clinicians and physician-scientists involved in this field.

## 1. Introduction

### An Interesting Evans Syndrome Case

A 6-month-old boy presented with pancytopenia, reticulocytopenia, a positive Coombs test, absent neutrophils with positive anti-neutrophil antibodies, and thrombocytopenia. He was diagnosed with ES. His bone marrow showed increased megakaryocytes and prominent myeloid development, in other words, hyperactive myeloid and megakaryocytic lineages, but also showed erythroid hypoplasia, consistent with pure red cell aplasia morphology, which itself can be an autoimmune pathology. IgM and IgA levels were elevated with normal IgG. Peripheral blood CD3+ T cells, the T cell CD4+/CD8+ ratio, CD19+ B cells, and CD56+ NK cells were within normal levels, and whole exome sequencing did not identify a known pathologic or potentially causative mutation for his pancytopenia.

Despite various treatment approaches, he developed severe thrombocytopenia, with single-digit platelet counts and multi-system severe bleeding, experienced recurrent infections, and was blood transfusion-dependent. He had several episodes of infections associated with severe neutropenia and requiring hospital admissions. Neutrophil recovery was seen following filgrastim therapy. Following rituximab therapy, B cells were eliminated completely in the peripheral blood without improvement in blood counts.

He was treated with bortezomib (1.3 mg/m^2^; a total of four every-3-day doses) 9 months after presentation and after failing oral prednisolone, intravenous immunoglobulin (IVIG), eltrombopag, rituximab, sirolimus, and intravenous high-dose methylprednisolone therapies. An increase in platelets was seen following the second dose of bortezomib; all blood counts normalized with reticulocytosis two weeks from the first dose. He was given IVIG supplementation since he had hypogammaglobulinemia and was completely B cell-depleted.

He had normal blood counts, serum immunoglobulin levels, and peripheral blood lymphocyte subsets, with a negative Coombs test and anti-neutrophil antibodies, 5 years after bortezomib therapy. This case was previously published [1].

Proteasomes are important cellular components that play a role in protein production, and plasma cells can be seen as immunoglobulin-producing factories. Bortezomib, a proteasome inhibitor, has activity against multiple myeloma, a plasma cell dyscrasia. This case emphasizes the potential role of proteasome inhibitors targeting plasma cells, perhaps as well as long-lived plasma cells (LLPCs), in immune cytopenia cases.

We will now discuss humoral immunity physiology with B cell and plasma cell development pertinent to this review, before focusing on the potential role of LLPCs in immune cytopenia further.

## 2. Humoral Immunity Physiology

### 2.1. B Cell Development

B cell development involves a sequence of stages where B cells mature and acquire functional capabilities in specialized organs commonly known as primary and secondary lymphoid organs. In B cell development, the primarily site is the red bone marrow in humans. After maturing in the primary lymphoid organs, B cells will eventually mount their immune responses in the secondary lymphoid organs, which are the lymph nodes, spleen, bone marrow, and other lymphoid tissues [2,3].

B cells arise from hematopoietic stem cells (HSCs) and will transform into an early lymphoid progenitor known as the common lymphoid progenitor (CLP), which ultimately gives rise to natural killer cells/dendritic cells on one the hand and, on the other, common lymphoid progenitor-2 (CLP-2), responsible for B cell lineage development [4]. The differentiation of the HSC into the immature B cell involves multiple phases. It starts off as the early pro-B cell, which undergoes variable, diversity, and joining (V(D)J) recombination, rearranging genes that encode the variable regions of the immunoglobulin heavy chains. When successful rearrangements occur, a functional heavy chain is made; this forms the pre-B cell receptor (pre-BCR). The pre-B cell, expressing a pre-BCR on the surface, undergoes further differentiation and proliferation. At this point, intact heavy μ chains are produced and exported to the cell surface in association with proteins that simulate surrogate light chains. The μ chains associated with the surrogate light chains form a complex expressed at the cell surface and transduce signals to stimulate the genes encoding functional light chains and cell proliferation. The expression of CD20 is now detectable at this stage. The final stage is the immature B cell, which after light-chain genes rearrange, express a complete B cell receptor (BCR). BCR expression is required for B cell survival in the peripheral tissues. Immature B cells express IgM on the surface as well as their known CD21 and CD22 markers [5,6,7].

Prior to migration into the periphery, B cells will undergo a selection process called central tolerance. At this time, autoreactive immature B cells undergo receptor editing upon binding self-antigens. If rearrangement of additional light chains fails, they are eventually marked for apoptosis [8]. Ultimately this process removes most self-reactive immature B cells that express polyreactive BCRs and antinuclear specificities [9].

**Figure 1:**
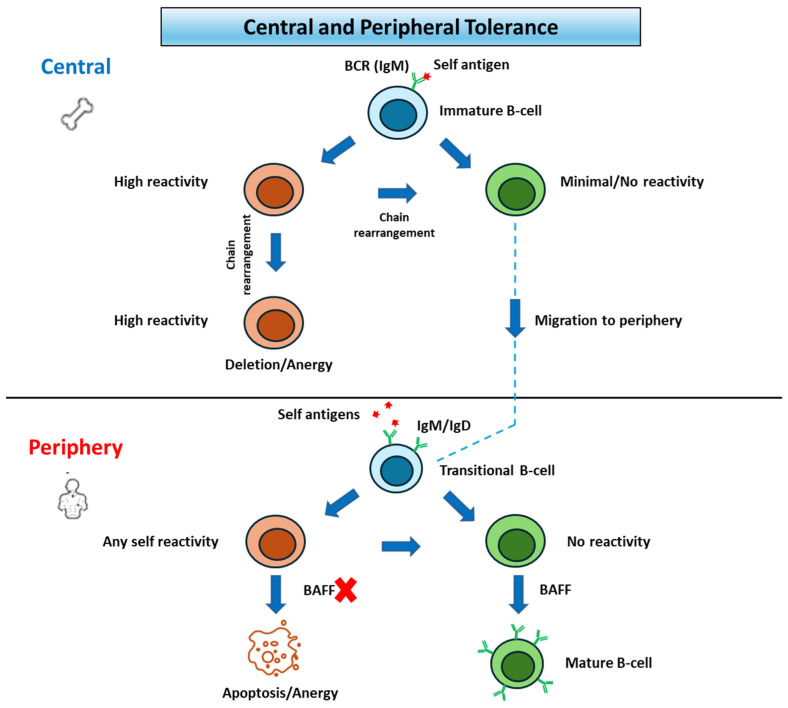
Immune tolerance development. Depiction of central and peripheral tolerance, which are checkpoints to capture and delete autoreactive B-cells. Central tolerance involves the immature B-cell and, if reactive, will undergo chain rearrangement to “fix” its reactivity to self-antigens, before undergoing apoptosis or anergy. Non-reactive cells then migrate to the periphery to undergo peripheral tolerance in the lymphoid tissues. Any reactivity will result in apoptosis or anergy in the deprivation of BAFF, while non-reactive cells will be able to mature. BCR: B cell receptor, BAFF: B cell-activating factor.

After migration, non-self-reactive immature B cells will move to the white pulp of the spleen and eventually become mature B-2 cells, expressing IgM+/IgD+, or become marginal zone (MZ) B cells. B-2 cells are activated in T-dependent immune responses and produce all subclasses of antibodies, as well as memory B cells with increased antibody affinity. MZ B cells on the other hand are found in the spleen and produce antibodies towards carbohydrate antigens. The process of maturation is strongly dependent on high concentrations of the cytokine B cell-activating factor (BAFF). Many self-reactive B cells that passed central tolerance undergo apoptosis in the spleen before they reach BAFF-rich follicles in a process called peripheral tolerance. This is contingent on activation of the B-cells by autoantigens, which arrest migration and ultimately undergo apoptosis due to BAFF-deprivation. Despite these checkpoints, which are displayed in Figure 1, autoantibodies have been found in healthy individuals, indicating that several self-reactive B cells escape tolerance mechanisms. Surviving mature B cells then reenter circulation and accrue in the follicles of secondary lymphoid tissues [3,10] (Figure 1).

#### 2.1.1. Antibodies and T Cell Help

Antibody affinity maturation is a process by which Ig genes undergo mutations leading to antibody molecules’ increased affinity for a specific antigen over time. Occurring in secondary lymphoid tissues, alterations in gene expression give rise to the germinal center (GC) reaction. B cells vie for antigens on dendritic cells by taking in and presenting them to T helper cells, which function to produce cytokines and further stimulate B cells, necessary for producing antibodies. There are multiple T helper subtypes that all play a role, most importantly T regulatory (Treg) cells, a subtype essential for preventing autoimmunity by suppressing T cells that have eluded the central or peripheral selection, and T follicular helper (Tfh) cells [4]. Tfh cells are necessary for sustained antibody production, as these cells interact with B cells through the secretion of several cytokines, including IL-4, IL-5, and IL-21, which are thought to be critical in B cell differentiation and antibody production [4,11]. Once an antigen is presented to the Tfh cell, it expresses the receptor with the greatest affinity in comparison to other T cells. Once interaction with the specific antigen occurs, somatic hypermutation will introduce point mutations in Ig genes. Low-affinity IgM is then produced, and further recombination will change IgM to either IgG-, IgA-, or IgE-specific antibodies, each with their own different functions. The final products are antibodies with a stronger affinity and specificity for that antigen [4,7,12,13,14]. This process is significant as it generates highly specific antibodies that will neutralize future pathogens or target antigens. It further ensures that subsequent immune responses will be more effective and possibly provide long-term immunity. Following an immune response, T and B cells differentiate into memory T cells and memory B cells, respectively. Memory T cells can respond quickly to re-exposure to the same antigen, whereas memory B cells can rapidly mature into plasma cells and produce high-affinity antibodies [10,15].

#### 2.1.2. Natural Autoantibodies

Natural autoantibodies are antibodies that recognize self-antigens in the absence of any immunization or infection. In contrast to antibodies produced against foreign antigens, natural autoantibodies in healthy individuals do not require any known prior exposure to the corresponding self-antigens. They are mainly of the IgM subclass, although IgG and IgA natural autoantibodies also exist [16,17]. The origin of natural autoantibodies is not well understood but likely arise during normal development and maturation of the immune system. The spleen is known to be a main site of autoantibody production by autoreactive B cells [18]. Elkon and Casali hypothesize that they are derived from B-1 cells, which make up 5% of the B cells in the body, and marginal zone B cells. Natural autoantibodies are commonly polyreactive; that is, they have the ability to bind to more than one self-antigen with low–moderate affinity, which may partly contribute to their wide protective action. They maintain immune homeostasis through their participation in clearing apoptotic cells and preventing the accumulation of potentially harmful self-antigens. Some natural autoantibodies can also recognize and neutralize microbial antigens, thus providing a first line of defense against infections until pathogen-specific antibodies are produced. Natural autoantibodies perform regulatory functions within the immune system and influence the activation and functions of other immune cells. However, alterations have been associated with various autoimmune diseases, suggesting that the dysregulation of their production or function could contribute to autoimmune pathology [16]. 

#### 2.1.3. Plasma Cell Development

The formation of plasma cells with resultant antibody production plays a role in how the immune system fights off infections and various obstacles it faces. This process of plasma cell development is carefully controlled to ensure the production of several antibodies tailored to target antigens. After stimulation with their self-specific antigen, mature B cells begin the process of further differentiation. Initially, B cells will undergo clonal expansion. Following this, a portion of B cells will then differentiate into plasma cells, which involves several key steps. This includes inducing transcription factors such as interferon regulatory factor 4 (IRF4) and B lymphocyte-induced maturation protein-1 (Blimp-1), which drive the differentiation of B cells into plasma cells. They also increase protein synthesis and develop plasma cell morphological changes crucial for antibody synthesis and secretion [4]. A cognate antigen, anti-B cell receptor antibody, complement receptors, or polyclonal stimulators like lipopolysaccharide may activate B cells, causing up-regulation and processing to the secretory form of immunoglobulin heavy-chain mRNA with resultant large quantities of immunoglobulin synthesis and the unfolded protein response to accommodate the massive quantity of secretory form production. This process is associated with increased formation of endoplasmic reticulum and Golgi in the cell cytoplasm. Activated B cells from either marginal zones or follicles can produce short-lived antibody-secreting cells. For the long-term survival of plasma cells, autophagy is a necessary process to eliminate some of the accumulated damage to the overworked ER. Bone marrow stromal cell signals contribute to plasma cell longevity [19]. Plasma cells excel at producing antibodies that target their stimulating antigen to mount a strong immune defense against the invading pathogen.

Plasma cells have varying lifespans. Some survive only for a few days to weeks, particularly during an acute immune response, while others can persist for months to years in specialized niches within the body, such as the bone marrow. The latter occurs by the interaction of a proliferation-inducing ligand (APRIL) and BAFF with B cell maturation antigen (BCMA), a receptor on plasma cells [3]. While IL-6 and APRILs are critical in maintenance, there is evidence that CD138 expression constitutes a survival advantage to more mature PCs [20]. Higher BCMA and lower BAFF expressions on PCs are critical in their maintenance, indicating the essential role of BCMAs in LLPC survival [21]. The development of LLPCs from antibody-secreting cells depends upon intrinsic factors such as cellular metabolism, anti-apoptotic capacity, and the regulation of autophagy and microenvironmental variables including cytokines, chemokines, and stromal cells [22].

Alongside the generation of plasma cells, some activated B cells differentiate into memory B cells [10]. These cells are responsible for mounting a quick and more effective immune response upon exposure to a specific antigen through maturing into plasma cells. Tfh cells are essential for the development and maintenance of long-lived plasma cells (LLPCs), as IL-21 production has specifically been shown to be important for inducing plasma cell differentiation in B cells. Tfh cells not only assist in the initial plasma cell differentiation of B cells but also further support the survival and maintenance of LLPCs [4,10].

#### 2.1.4. Long Lived Plasma Cells

Long-lived plasma cells are a specialized subset of plasma cells, expressing the markers CD38 and CD138, that play a crucial role in maintaining long-term humoral immunity. As mentioned above, LLPCs are the products of activated B cells that have undergone differentiation into plasma cells in response to antigens. They develop through=gh the expression of transcription factor Blimp-1, and their prolonged survival is due to the presence of IL-16 [4]. LLPCs are typically produced in secondary lymphoid organs and can migrate to specialized niches within the body, typically in the bone marrow (80–90%), where they are able to survive for decades [23]. This is attributed to extended production of IgG-type antibodies within the niche that can mount a permanent defense against the original antigen [4].

There are many roles in which LLPCs are significant. For instance, they are crucial for maintaining protective antibody levels in circulation and mucosal surfaces. They provide a robust antibody response upon re-exposure to the same antigen, contributing to immunological memory. Their sustained antibody production helps in preventing reinfection and contributing to vaccine-induced immunity. Dysregulation of LLPC development or function can contribute to autoimmune diseases, where autoantibodies are produced against self-antigens. Understanding LLPC biology is crucial for developing strategies to enhance vaccine responses or suppress harmful antibody production in autoimmune diseases.

**Figure 2:**
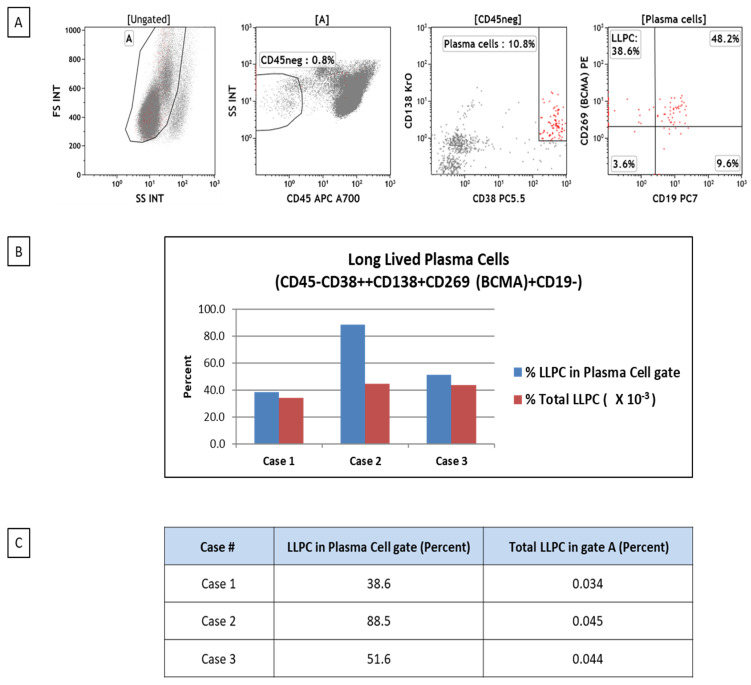
Flow cytometric analysis of bone marrow plasma cells. Flow cytometry dot plots demonstrating the gating strategy of plasma cells and long-lived plasma cell subset (LLPCs) in Case 1. Based on forward-scatter and side-scatter characteristics, live cell gate A is analyzed in the CD45/side-scatter plot to gate on the CD45-negative subset, which is further analyzed for CD138 and CD38 expression to identify the percentage of plasma cells (CD138+CD38high+). The plasma cells are then analyzed for CD269(BCMA) and CD19 expression to display the percent population of CD45–CD138+CD38high+CD269 (BCMA)+CD19– LLPCs within the plasma cell subset (rightmost dot plot). The majority of LLPCs are seen piling up at the axis in the dot plot (**A**). Additionally, the bar graph (**B**) and table (**C**) display percent populations of LLPCs in plasma cell gates and percent total populations of LLPCs in live bone marrow mononuclear cells (gate A) in three cases of bone marrow failure.

LLPC can be detected and quantified using flow cytometry as a useful tool in studying plasma cell biology in different clinical conditions. We have been investigating the frequencies of plasma cells and LLPCs in patients with bone marrow failure. LLPCs are characterized by the CD45–/CD38-high/CD138+/BCMA+/CD19– phenotype (Figure 2).

### 2.2. Humoral Immunity Deviation in Autoimmunity

LLPCs are among the major players in humoral autoimmunity. In autoimmune diseases, tissues and cells are destroyed due to the production of autoantibodies that lead to damage and inflammation. Accordingly, LLPCs possess the ability to produce large amounts of antibodies and autoantibodies. Their prolonged survival allows them to continuously produce autoantibodies, thus contributing to the chronicity of the autoimmune reaction. There have been cases of increased frequencies of CD38+ plasma cells identified in patients with anti-neutrophil cytoplasmic autoantibody (ANCA)-associated vasculitis (AAV) and in patients with SLE. Patients with established rheumatoid arthritis were found to have larger fractions of CD38+ cells within the synovium compared to healthy individuals [24].

### 2.3. Long Lived Plasma Cells in Immune Cytopenia

LLPCs may play a role in immune cytopenia, similarly to how they are involved in autoimmunity in general. Immune cytopenias are typically acquired conditions, usually characterized by the immune destruction of red blood cells, platelets, neutrophils, or combination of more than one type. They include autoimmune hemolytic anemia (AIHA), immune thrombocytopenia (ITP), and autoimmune neutropenia (AIN). In some cases, more than one can occur at the same time, indicating a disease known as Evans syndrome (ES) [25]. In immune cytopenia, it is the autoantibodies that specifically target the cells produced by plasma cells [18]. Further, it has been hypothesized that LLPCs specifically are a driving mechanism in patients with ITP or AIHA, after residual CD19+/CD138+ was found in splenocytes approximately 6 months after administration of rituximab, a CD20-depleting therapy [23]. The spleen is considered the primary site of autoantibody production by autoreactive B cells and platelet destruction in ITP; a significant proportion of ITP patients possess anti-integrin αIIbβ3 IgG autoantibodies leading to platelet opsonization and phagocytosis. Failure of rituximab therapy causing B cell depletion and splenectomy in some ITP cases suggests that autoreactive, CD20-depletion-resistant, IgG-secreting cells reside in other anatomical locations. Furthermore, daratumumab therapy targeting CD38 on plasma cells had variable clinical responses with the persistence of autoreactive high-affinity anti–αIIbβ3-secreting plasma cells in the bone marrow [18]. It is also believed that there is dysfunction or imbalance in regulatory T cells that ultimately leads to inadequate control over autoreactive B cell responses [26].

**Figure 3:**
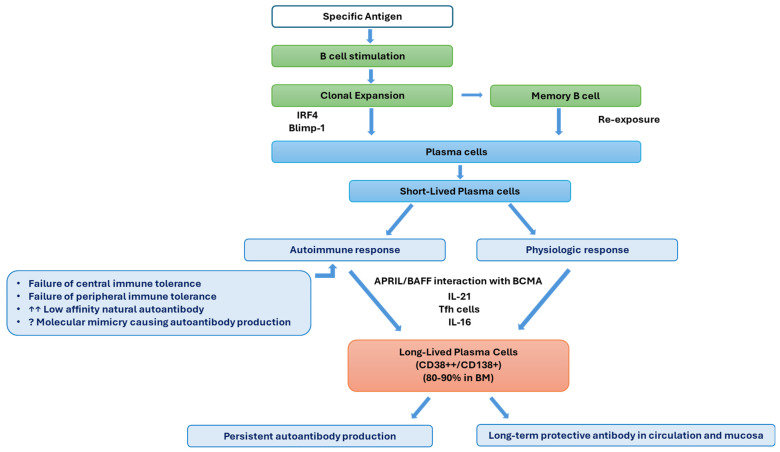
Development of plasma cells and long-lived plasma cells (LLPCs) and their potential role in autoimmune cytopenias. While LLPCs have a central role in maintaining antibody levels against several foreign immunogens, the deviation of the reaction to target self-antigens leads to autoantibody production. There are several proposed mechanisms in the development of autoreactive plasma cells, including the failure of central or peripheral immune tolerance, uncontrolled secretion of low-affinity polyreactive natural antibodies with potential affinity maturation, and autoantibody production due to molecular mimicry. IRF4: interferon regulatory factor 4, Blimp-1: B lymphocyte-induced maturation protein-1, IL-21: interleukin 21, Tfh: T follicular helper, IL-16: interleukin 16, APRIL: a proliferation-inducing ligand, BAFF: B cell-activating factor, BCMA: B cell maturation antigen, BM: bone marrow.

Although negative selection checkpoints mediate the elimination of high-affinity, self-reactive lymphocytes, there is strong evidence that low-affinity autoreactive cells persist and contribute significantly to immune dysregulation. Organ-specific autoantibodies in certain autoimmune diseases do strongly suggest stimulation of autoantibodies by inflammation. However, in systemic autoimmune diseases such as SLE or immune cytopenias, the origin of autoantibodies is not as clear due to their non-specificity and effects on multiple target organs, suggesting some similarity to natural polyreactive antibodies [16]. Plasma cell development and the potential role of LLPCs are depicted in Figure 3.

### 2.4. Treatment

The treatment of both autoimmune diseases and immune cytopenias can be difficult as they are chronic and, at times, resistant to available first-line therapeutic options, requiring a broad range of alternative therapies [25]. Further, LLPCs are relatively resistant to conventional therapies that target rapidly dividing cells, likely due to their lack of proliferative activity and their location in protective niches, i.e., within the bone marrow, which makes them less accessible. Understanding the biology of LLPCs in autoimmunity offers potential therapeutic avenues. First-line therapies for different types of immune cytopenias are well laid out and cases are often managed using corticosteroids. However, there is no consensus regarding the proper approach of second- and even third-line therapies. The known side effects of steroids have sparked the search in developing steroid-sparing therapeutic regimens. Rituximab (anti-CD20 monoclonal antibody) is a common first- or second-line medication for some autoimmune disease and immune cytopenia cases. However, paradoxically, B cell depletion therapy stimulates an adaptation of splenic short-lived plasma cells that leads to their reprogramming into long-lived spleen cells in patients with ITP, causing failure of rituximab [18]. Additionally, as LLPCs are characterized by the CD138+/CD19– phenotype, and some lack CD20 expression, rituximab may not be efficient in eliminating autoantibodies producing entire plasma cell populations.

Second-/third-line therapies are far broader and include mycophenolate mofetil, thrombopoietin receptor agonists (eltrombopag and romiplostim), mTOR inhibitors (sirolimus and everlimus), azathioprine, cyclosporin, and other immunosuppressive medications. Of note, some of these drugs are off-label for indication and/or for age. Also, there is a probable link between the mortality of patients who received more second- and third-line treatments in certain autoimmune diseases [27]. The final step of treatment, in some cases, is splenectomy, but it is generally avoided due to the risk of post-splenectomy complications [25]. Strategies to selectively target plasma cells and LLPCs, inhibiting their survival and antibody production, are actively being investigated to improve treatment outcomes in autoimmune diseases.

Several novel immunologic therapeutic modalities targeting B, T, and plasma cells have been introduced for the treatment of autoimmune diseases, including chimeric antigen receptor-T (CAR-T) cell therapies [24,28]. CAR-T cells directed at CD19 can potentially eliminate tissue resident-B cells in addition to ones in the fluid phase, resulting in clinical remission. Daratumumab, an anti-CD38 monoclonal antibody used in multiple myeloma treatment, preferentially targets plasmablasts, short-lived plasma cells, and LLPCs. Daratumumab has been shown to be successful in refractory immune cytopenia [29,30,31]. Since LLPCs express CD138, CAR-T cell therapy targeting CD138, such as indatuximab, may be an option in the future. Furthermore, recent evidence points to the feasibility of BCMA-targeting T cell-engager therapies in autoimmune disorders [32].

### 2.5. Cancer and Immune Cytopenia

There is a relationship found between immune cytopenia and cancer. On one hand, immune cytopenia can occur as a complication of various malignancies, with a prevalence of up to 10%. Both Hodgkin and non-Hodgkin lymphomas can present with ITP, likely due to autoimmune response. It may be found shortly before diagnosis of lymphoma or during workup. ITP is a frequent complication of chronic lymphocytic leukemia, with reports up to 2.9% [33]. Interestingly, different types of immune cytopenias have been reported in a variety of malignant neoplastic conditions, many of which happen to be hematolymphopoetic in origin, including multiple myeloma, chronic lymphocytic leukemia, myelodysplastic syndrome, chronic myelomonocytic leukemia, lymphomas, and leukemias [34,35,36]. This observation may reflect a relationship between lymphoplasmocytic proliferations and the development of autoimmune phenomena. Though, ITP has been reported in association with various types of cancer as well [37].

On the other hand, immune cytopenia itself may directly increase the risk of developing cancer, with the belief that it is due to persistent inflammation, bone marrow dysfunction, and immune dysregulation. Furthermore, immunosuppressive drug treatment may also contribute to the development of malignancies. Studies have shown that patients with autoimmune cytopenias have an increased risk of malignancies compared to the general population, with a nearly 40% increase. These studies have linked ITP, rheumatoid arthritis, and Sjogren syndrome to Hodgkin and non-Hodgkin lymphoma and myeloproliferative neoplasms. Further, patients with ITP have a higher risk of being diagnosed with solid gastro-intestinal (i.e., liver), skin, and hematological cancers [38].

### 2.6. Genetics and Epigenetics of Immune Cytopenia

The causes of immune cytopenia and autoimmunity are considered multifactorial, with genetic, epigenetic, and environmental factors contributing to defects in the immune system that lead to autoimmune reactions [16,39,40]. In a retrospective analysis, 15.9% of newly diagnosed and persistent ITP and 29.8% of chronic ITP cases were found to have familial autoimmunity [41]. Several mutations in various immune response-associated genes have been reported in ES [42].

Circulating antibody-secreting cells have a decreased nucleus/cytoplasm ratio, with increased endoplasmic reticulum and numbers of mitochondria compared to bone marrow LLPCs. While MCL1, BCL2, and BCL-XL are up-regulated to help with survival, pro-apoptotic HRK1, CASP3, and CASP8 are down-regulated. Pro-apoptotic gene loci are less accessible in LLPCs, whereas only BCL2 is concordant in gene up-regulation and loci accessibility. In vitro evidence demonstrated that blood antibody-secreting cells undergo morphological, transcriptional, and epigenetic changes to mature into LLPCs in the bone marrow niche [43].

Joyner Epigenetics encompasses cellular modifications that lead to alterations in gene expression or cellular phenotype without any changes in the DNA sequence. Epigenetic research seeks to identify the ways in which environmental agents or other exogenous factors can impact gene activity and gene expression and the degree to which such modifications might be passed on to subsequent generations. These modifications, which include DNA methylation, non-coding RNA, and histone modification, are required for correct development, cell differentiation, and maintenance of the cellular identity of hematopoietic stem cells into different immune cell types. For instance, during B cell activation and differentiation, the epigenome undergoes a remarkable shift that involves active events of various types [44]. Dysregulation in these processes leads to impaired immune function and contributes to disease manifestations.

Plasma cells differentiate through the tightly regulated action of transcription factors and surrounding environmental influences. The majority of the detailed mechanisms of epigenetic events that sustain plasma cell identity, and their functions, remain to be defined [44]. What is known includes DNA methylation, which controls the expression of genes critical for plasma cell differentiation and function. Further, histone acetylation and methylation have roles in the accessibility of chromatin regions associated with plasma cell-specific genes [45].

For instance, increased histone acetylation is associated with active transcription of genes involved in plasma cell function. Epigenetic modifications influence the binding of transcription factors that drive plasma cell development. Examples include highly specialized plasma cell differentiation, which requires key transcription factors, including but not limited to BLIMP-1 and X-box binding protein (XBP-1), whose expression is controlled by epigenetic modifications [46]. Long-lived plasma cells residing in the bone marrow depend on the stable epigenetic environmental influences for continuous functionality; this stability is achieved through epigenetic silencing. LLPCs often have epigenetic modifications that silence genes not needed for their function, helping sustain their longevity and protect against apoptosis. Specific chromatin remodeling events ensure that essential genes for plasma cell survival and antibody production remain active, while others are repressed. The microenvironment of the bone marrow involves signals that affect the epigenetic landscape of long-lived plasma cells. The interaction between stromal cells, cytokines, and other factors modifies the epigenetic regulation of genes necessary for the maintenance and survival of plasma cells [47].

Epigenetic dysregulation has been implicated in autoimmune diseases and immune cytopenias, among other conditions. These modifications are the crucial step in immune tolerance, impairment, and pathogenesis, since these pathways play a pivotal role in various functions of apoptosis and inflammation [26]. For instance, studies have shown global H3K9 hypomethylation in T cells in patients with ITP, or in other cases, have shown high methylation of forkhead box P3 (FOXP3), a player in the development of T reg cells, which results in lower FOXP3 gene expression [26,48].

Research into epigenetics not only allows us to understand diseases but has led to the development of interventions that can target epigenetic mechanisms. We know, for instance, that the activation of oncogenes or the silencing of tumor suppressor genes contributes to cancer development. Therefore, one of the biggest results of epigenetic studies has been methyltransferase inhibitors and histone deacetylase inhibitors, which are used in the treatment of certain cancers. Most recently, there have been discoveries of novel therapeutic options in autoimmunity and even ITP that could not only treat but potentially reverse epigenetic changes [48].

Currently, it has been challenging to find a link between genetic conditions that are more prone to LLPC production, though there is some overlap in similar genetic variants. For instance, there is a possibility of critical roles for STAT3 and NF-kB as keys in LLPCs that reinforce their survival and longevity, and we know that there have been findings of STAT3 and NF-kB mutations in ES patients [49]. However, there have been no correlations made at this time. A study showed that among patients with pediatric ES, those without a known gene defect had similar immune abnormalities compared to patients with known defects. This, in some respects, postulates a possible genetic component that has not yet been discovered or other epigenetic mechanisms. Therefore, determining specific genetic abnormalities prone to developing LLPCs warrants further investigation [11].

Combining epigenetic data with genomic and transcriptomic analyses could provide a more comprehensive understanding of how epigenetic changes interact with genetic factors to influence immune cell function and contribute to immune cytopenias. Further, with increasing availability of methods for detecting epigenomic changes, strategies for genome-wide epigenetic analysis of different immune cell subsets could be rapidly developed [44].
